# Amyloid PET in European and North American cohorts; and exploring age as a limit to clinical use of amyloid imaging

**DOI:** 10.1007/s00259-015-3115-5

**Published:** 2015-07-02

**Authors:** Konstantinos Chiotis, Stephen F. Carter, Karim Farid, Irina Savitcheva, Agneta Nordberg, for the Diagnostic Molecular Imaging (DiMI) network and the Alzheimer’s Disease Neuroimaging Initiative

**Affiliations:** Department of NVS, Center for Alzheimer Research, Translational Alzheimer Neurobiology, Karolinska Institutet, Stockholm, Sweden; Wolfson Molecular Imaging Centre, Institute of Brain, Behaviour and Mental Health, University of Manchester, Manchester, UK; APHP, Hotel-Dieu Hospital, Department of Nuclear Medicine, Paris, France; Department of Radiology, Karolinska University Hospital Huddinge, Stockholm, Sweden; Department of Geriatric Medicine, Karolinska University Hospital Huddinge, Stockholm, Sweden

**Keywords:** Alzheimer’s disease, Amyloid-PET, [11C]PIB, [18F]Florbetapir, Age, Diagnosis

## Abstract

**Purpose:**

Several radiotracers that bind to fibrillar amyloid-beta in the brain have been developed and used in various patient cohorts. This study aimed to investigate the comparability of two amyloid positron emission tomography (PET) tracers as well as examine how age affects the discriminative properties of amyloid PET imaging.

**Methods:**

Fifty-one healthy controls (HCs), 72 patients with mild cognitive impairment (MCI) and 90 patients with Alzheimer’s disease (AD) from a European cohort were scanned with [11C]Pittsburgh compound-B (PIB) and compared with an age-, sex- and disease severity-matched population of 51 HC, 72 MCI and 84 AD patients from a North American cohort who were scanned with [18F]Florbetapir. An additional North American population of 246 HC, 342 MCI and 138 AD patients with a Florbetapir scan was split by age (55–75 vs 76–93 y) into groups matched for gender and disease severity. PET template-based analyses were used to quantify regional tracer uptake.

**Results:**

The mean regional uptake patterns were similar and strong correlations were found between the two tracers across the regions of interest in HC (ρ = 0.671, *p* = 0.02), amyloid-positive MCI (ρ = 0.902, *p* < 0.001) and AD patients (ρ = 0.853, *p* < 0.001). The application of the Florbetapir cut-off point resulted in a higher proportion of amyloid-positive HC and a lower proportion of amyloid-positive AD patients in the older group (28 and 30 %, respectively) than in the younger group (19 and 20 %, respectively).

**Conclusions:**

These results illustrate the comparability of Florbetapir and PIB in unrelated but matched patient populations. The role of amyloid PET imaging becomes increasingly important with increasing age in the diagnostic assessment of clinically impaired patients.

**Electronic supplementary material:**

The online version of this article (doi:10.1007/s00259-015-3115-5) contains supplementary material, which is available to authorized users.

## Introduction

Alzheimer’s disease (AD) pathology is characterised by the accumulation of amyloid-beta (Aβ) plaques, neurofibrillary tangles and neuronal degeneration [1, 2]. The use of positron emission tomography (PET) with amyloid-specific radiotracers has enabled in vivo imaging of fibrillar Aβ plaques; [11C]Pittsburgh compound-B (PIB) was the first generation of these tracers [3]. The short half-life of [11C] (~20 min) led to the development of [18F] derivatives (half-life ~110 min) [4]. The longer half-life of [18F] negates the need for on-site cyclotron and radiochemistry facilities, as [18 F] tracers can be produced centrally and delivered to geographically dispersed sites, thus enabling their clinical use. [18F]Florbetapir (Florbetapir) was the first [18 F] radiotracer specific for Aβ to receive approval from both the U.S. Food and Drug Administration (FDA) (2012) and the European Medicines Agency (2013) for assessing the presence of Aβ pathology in individuals with cognitive decline. These advances in molecular imaging led to a proposed revision of the diagnostic criteria for AD, in which amyloid PET plays an important role [5].

Autopsy studies of individuals who were scanned prior to death using PIB or Florbetapir have confirmed that both radiotracers bind selectively to fibrillar Aβ [6–9]. Moreover, in vivo studies have demonstrated the comparability of these two amyloid radiotracers within the same patient population [10–12]; however, it is uncertain how comparable PIB and Florbetapir are between different populations.

The ages of individuals who present to a memory clinic with cognitive deficits typically span a wide continuum and it is not yet completely clear what impact age has on the discriminative ability of amyloid imaging. It has been demonstrated that cognitively normal adults aged >60 years can possess pathological brain levels of fibrillar Aβ (i.e. they are amyloid positive) [13–19]. The number of amyloid-positive, cognitively normal individuals increases with age from ~6 % of those aged between 55 and 60 years to >30 % of those aged 80 years or more [17]. It is imperative to understand whether these individuals are in a high-risk pre-AD state or are experiencing normal age-related brain changes. Additionally, much uncertainty still exists about the relative Aβ loads of AD patients in different age groups. Most research arbitrarily splits patients into two subtypes based on a cut-off point at 65 years of age, namely early-onset and late-onset AD (EoAD and LoAD, respectively). Several *post mortem* studies have demonstrated significantly greater Aβ burden in patients with EoAD than in those with LoAD [20–22], but PET studies have failed to give consistent results [23–25]. It is clear that understanding the impact of age on amyloid PET results is an important issue, regardless of whether individuals present at an asymptomatic or advanced disease state.

The purpose of this investigation was, therefore, twofold: 1) to validate cross-radiotracer comparisons (PIB vs Florbetapir) in unrelated but matched patient populations and determine the comparability of amyloid PET data; and 2) to examine the distribution of amyloid-positive brain scans in different age groups, establish how age affects the discriminative power of amyloid PET imaging in those of advanced age (where >30 % of cognitively normal adults can be amyloid positive) and establish whether apolipoprotein ε4 (ApoE4) differentially mediates the Aβ load in AD patients at different ages. To achieve these goals, data were used from two well-characterised data sets that have both clinical and amyloid PET data available. PIB data were obtained from the Diagnostic Molecular Imaging (DiMI) consortium and Florbetapir data were downloaded from the Alzheimer’s Disease Neuroimaging Initiative (ADNI) site.

## Materials & methods

### DiMI consortium

Some of the data used in the preparation of this article were obtained from the European Network of Excellence on DiMI, which was funded by the European Union (EC-FP6), launched in 2005 and ended in 2010. The goal of the DiMI consortium, which comprised six groups from five European countries, was to retrospectively collect PET data from five European centres in order to determine the role of amyloid imaging (using PIB as tracer) in a large population of mild cognitive impairment (MCI) and AD patients [26].

### ADNI

The remaining data used in the preparation of this article were obtained from the ADNI database (adni.loni.usc.edu). The ADNI was launched in 2003 by the National Institute on Aging (NIA), the National Institute of Biomedical Imaging and Bioengineering (NIBIB), the FDA, private pharmaceutical companies and non-profit organisations, as a $60 million, 5-year public-private partnership. The primary goal of ADNI has been to test whether serial magnetic resonance imaging (MRI), PET, other biological markers, and clinical and neuropsychological assessment can be combined to measure the progression of MCI and early AD. Determination of sensitive and specific markers of very early AD progression is intended to aid researchers and clinicians to develop new treatments and monitor their effectiveness, as well as lessen the time and cost of clinical trials.

The principal investigator in this initiative is Michael W. Weiner, MD (VA Medical Center and University of California – San Francisco). ADNI is the result of the efforts of many co-investigators from a broad range of academic institutions and private corporations, and individuals have been recruited from over 50 sites across the USA and Canada. The initial goal of the ADNI was to recruit 800 individuals but ADNI has been followed by ADNI-GO and ADNI-2. To date these three protocols have recruited over 1500 adults, ages 55 to 90, to participate in the research, consisting of cognitively normal older individuals, people with early or late MCI, and people with early AD. The follow-up duration of each group is specified in the protocols for ADNI-1, ADNI-2 and ADNI-GO. Individuals originally recruited for ADNI-1 and ADNI-GO had the option to be followed in ADNI-2. For up-to-date information, see www.adni-info.org.

### Study population

Data included in this study were derived from two patient cohorts, as follows.

### DiMI cohort – [11C]PIB

Data were collected from a population comprising 51 healthy controls (HCs), 72 MCI patients and 90 AD patients from the DiMI PIB consortium [26]. The AD patients fulfilled the NINCDS-ADRDA criteria for probable AD [27] and the DSM-IV criteria for dementia of AD type [28], whereas the MCI patients fulfilled the Petersen criteria [29]. The patients were recruited from five European research centres for AD (Technische Universität München, Munich, Germany; Katolieke Universiteit Leuven, Leuven, Belgium; Imperial College London, London, UK; Karolinska Institutet, Stockholm, Sweden; and Turku PET Centre, University of Turku, Finland) and completed at least one PIB scanning session. More details can be found in Nordberg et al. [26]. Data regarding the education and ApoE4 status of the participants are reported in Table 1.

**Table 1:** Demographics for the between-population (DiMI vs ADNI), cross-radiotracer ([11C]PIB vs [18F]Florbetapir) comparison

	DiMI ([11C]PIB) (n = 213)			ADNI ([18F]Florbetapir) (n = 207)		
	HC	MCI	AD	HC	MCI	AD
n	51	72	90	51	72	84
Age (years)	67.4 ± 6.3	67.5 ± 8.1	69.9 ± 8.2	70.6 ± 3.1	71.8 ± 2.3	69.0 ± 5.3
Gender (m/f)	22/29	37/35	42/48	22/29	37/35	42/42
Education data available, n (mean years ± SD^a^)	27 (13.2 ± 2.2)	42 (12.9 ± 3.2)	69 (12.0 ± 3.1)	51 (16.8 ± 2.7)	72 (16.1 ± 2.6)	84 (15.8 ± 2.6)
ApoE4 status available, n (E4 non-carriers, carriers)	31 (21, 10)	59 (25, 34)	80 (21, 59)	51 (36, 15)	70 (36, 34)	84 (18, 66)
MMSE data available, n (mean score ± SD)	43 (29.2 ± 1.1)	72 (27.1 ± 2.0)	90 (23.8 ± 3.1)	51 (29.2 ± 0.9)	72 (27.5 ± 1.8)	84 (23.1 ± 2.3)
Amyloid positive, n (%)	5 (10)	46 (64)	82 (91)	9 (18)	31 (43)	71 (85)

Amyloid positivity has been defined as a composite neocortical ratio (CCTXR) value above the cut-off point of 1.42 for [11C]PIB and 1.34 for [18F]Florbetapir. (AD: Alzheimer’s disease; SD: standard deviation)

^a^ The years of education for the individuals scanned with [11C]PIB were significantly fewer than the years of the education for the individuals scanned with [18 F]Florbetapir in the HC (*p* < 0.001), MCI (*p* < 0.001) and AD (*p* < 0.001) groups

### ADNI cohort – [18F] Florbetapir

Data for a population of 916 individuals who had had at least one Florbetapir scan were downloaded from the ADNI database. The AD patients fulfilled the NINCDS-ADRDA criteria [27] whereas the MCI patients had a clinical dementia rating of 0.5, abnormal memory function as documented on the Logical Memory II subscale, and were not demented [30]. After exclusion of 11 individuals without MRI scans or with poor-quality Florbetapir scans, we composed two groups from the remaining participants. First, age, mini-mental state examination (MMSE) score and gender distribution were randomly matched in a relatively young group of 51 HC, 72 MCI and 84 AD patients to the participants of the DiMI cohort. Second, the total ADNI population was split into younger (aged 55-75 y) and older (aged 76-83 y) subgroups. Randomised matching of these subgroups for MMSE score and gender distribution (HC, and MCI or AD patients) resulted in 363 individuals in each age subgroup. 159 individuals from the ADNI database appeared in both analyses. From the total of 726 participants, 715 had available data regarding their ApoE4 carrier status.

### Image analysis

#### [11C]PIB

All of the PIB data were processed in accordance with the methodology outlined in Nordberg et al. [26]; PIB data were summed between 40 and 60 min after injection of the tracer for each of the 213 participants. All available PET data sets (n = 213) were non-linearly spatially normalised to a population-specific PIB-PET template based on the mean PIB data from the 151 individuals who had MRI data available. A binarised grey matter (GM) anatomical mask was generated and multiplied by a standard digital atlas [31] to create 12 bilateral anatomically defined GM regions of interest (ROI) (temporal lobe, frontal lobe, occipital lobe, parietal lobe, insular lobe, anterior cingulate, posterior cingulate, caudate nucleus, putamen, thalamus, hippocampus, and parahippocampal gyrus) in Montreal Neurological Institute (MNI) space. A cerebellar GM region was used as reference for each 40–60 min PIB image in order to create standardized uptake value ratio (SUVR) images for each individual (based on the median cerebellar uptake). A composite neocortical ratio (CCTXR) was calculated for each individual, resulting in a weighted average for the frontal, parietal and basal/lateral temporal regions. More details regarding the applied methodology can be found in the original publication [26].

#### [18 F]Florbetapir

Raw Florbetapir frames acquired between 50 and 70 min after injection of the tracer were downloaded from the ADNI database and summed. The 207 Florbetapir images used in the comparison with PIB data had an accompanying MRI image that was used for the spatial normalisation of the respective integral co-registered PET images to MNI space, similarly to the method used for the PIB data. An average Florbetapir image was generated from these 207 images and used as a sample-based Florbetapir-PET template in MNI space. All raw Florbetapir images used in this project (*n* = 774) were subsequently spatially normalised to MNI space with the use of this template. A whole cerebellum region (based on the mean cerebellar uptake) was used as a reference for all 50-70 min Florbetapir images as it displayed lower variance of the HCs and larger effect size between HC and AD patients in comparison to the cerebellar GM region (Supplementary Table 1). The same anatomical mask as that used for the PIB data was applied to the Florbetapir data in order to define GM ROIs on the standard digital atlas, resulting in the same 12 bilateral GM ROIs as well as a CCTXR, as described above.

The pre-processing of the images (i.e., segmentation, co-registration as well as spatial normalisation steps) for both tracers was performed using SPM5 (Functional Imaging Laboratory, Wellcome Department of Imaging Neuroscience, UCL, London).

### Visual assessment of [18 F]Flurodeoxyglucose (FDG) scans

The ADNI database FDG-PET (30–60 min) images from the AD patients in both age groups whose Florbetapir scans were negative for amyloid using the quantitative method, according to the cut-off point described below, were visually assessed for the presence of an AD-like pattern of hypometabolism [32] by two independent, experienced nuclear medicine specialists (raters) who were blinded to the clinical and demographic backgrounds of the individuals.

### Statistical methods

Differences between the groups were assessed with analysis of variance (ANOVA) and chi-squared tests. Two-way ANOVA was performed in order to evaluate the influence of the factors, age group and ApoE4 carrier status, on regional Florbetapir uptake. Two levels for age group (55–75 and 76–83 y subgroups) and two for ApoE4 status (ApoE4 carriers and non-carriers) were set. Correlations were examined with the Spearman rank correlation coefficient (ρ). Receiver operating characteristic (ROC) analysis between HC and AD patients was used to identify the points on the curve closest to (0, 1), as optimum cut-off points. Sensitivity and specificity were calculated. Cohen’s kappa (κ) [33] and Cohen’s d [34] were calculated. The expectation-maximisation algorithm for mixture models was used to identify the underlying parameters of the studied sample and to cluster the individuals into subpopulations according to their probability distributions within the overall population [35].

All statistical analyses and graphical representations were carried out using SPSS 22.0 for Mac OS X, except for the mixture model analysis, which was carried out in XLSTAT 2014.2.03 for Microsoft Excel for Mac 2011.

## Results

### Comparison between PIB and Florbetapir

#### Demographics

The HC, MCI and AD patients from both cohorts were matched with regard to their age, gender and general cognitive status (MMSE). There were no differences in distribution of the ApoE4 allele between the two cohorts across the different diagnostic groups. The individuals from the ADNI cohort were significantly more educated than those from the DiMI cohort (Table 1).

#### Uptake characteristics

**Fig. 1:**
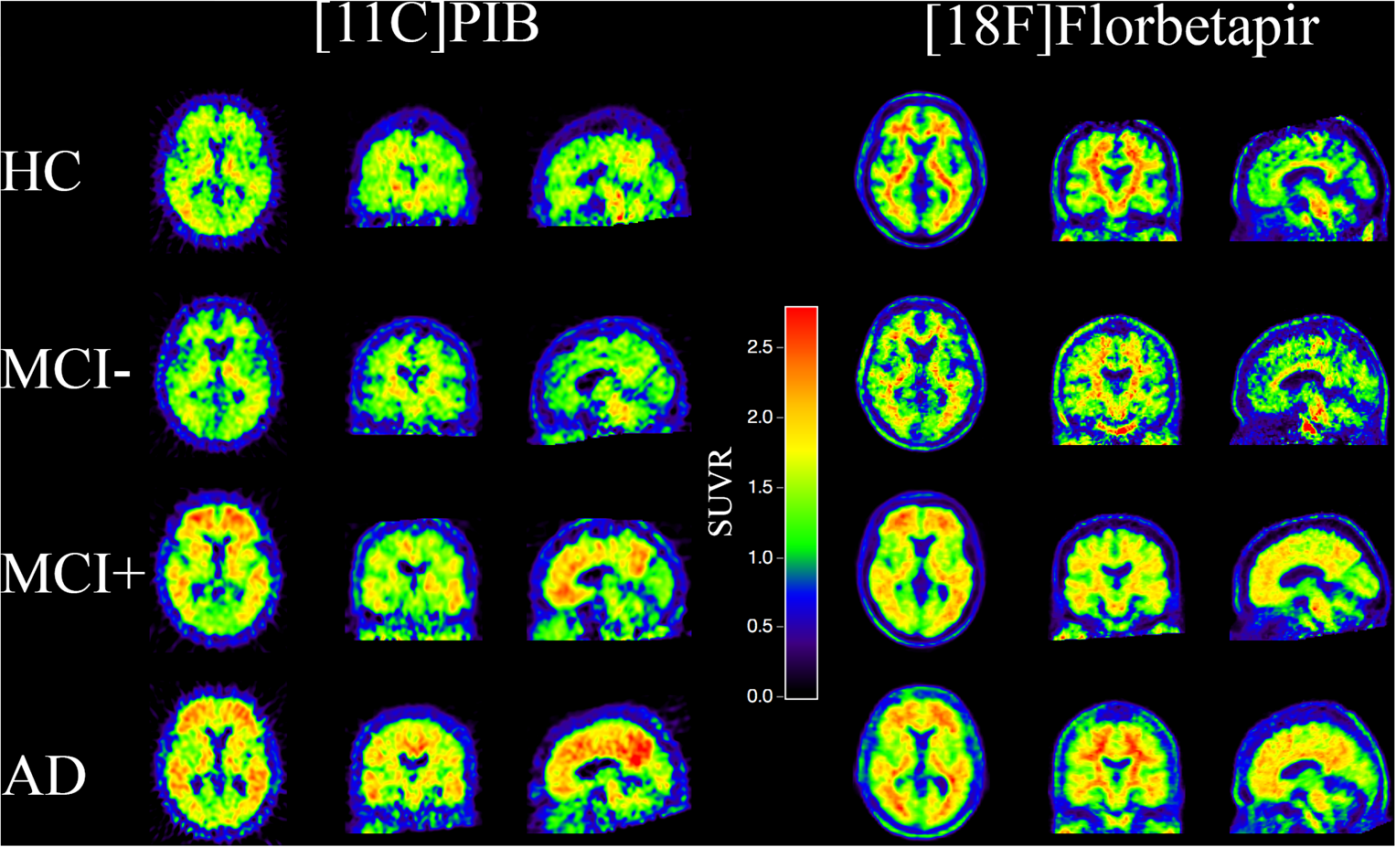
Typical examples of individual SUVR images from HC, and MCI amyloid-negative (MCI-), MCI amyloid-positive (MCI+) and AD patients matched for age, gender and MMSE. [11C]PIB scans are on the left and [18F]Florbetapir scans are on the right. Amyloid positivity has been defined as a CCTXR value above the cut-off points of 1.42 for [11C]PIB and 1.34 for [18F]Florbetapir

**Fig. 2:**
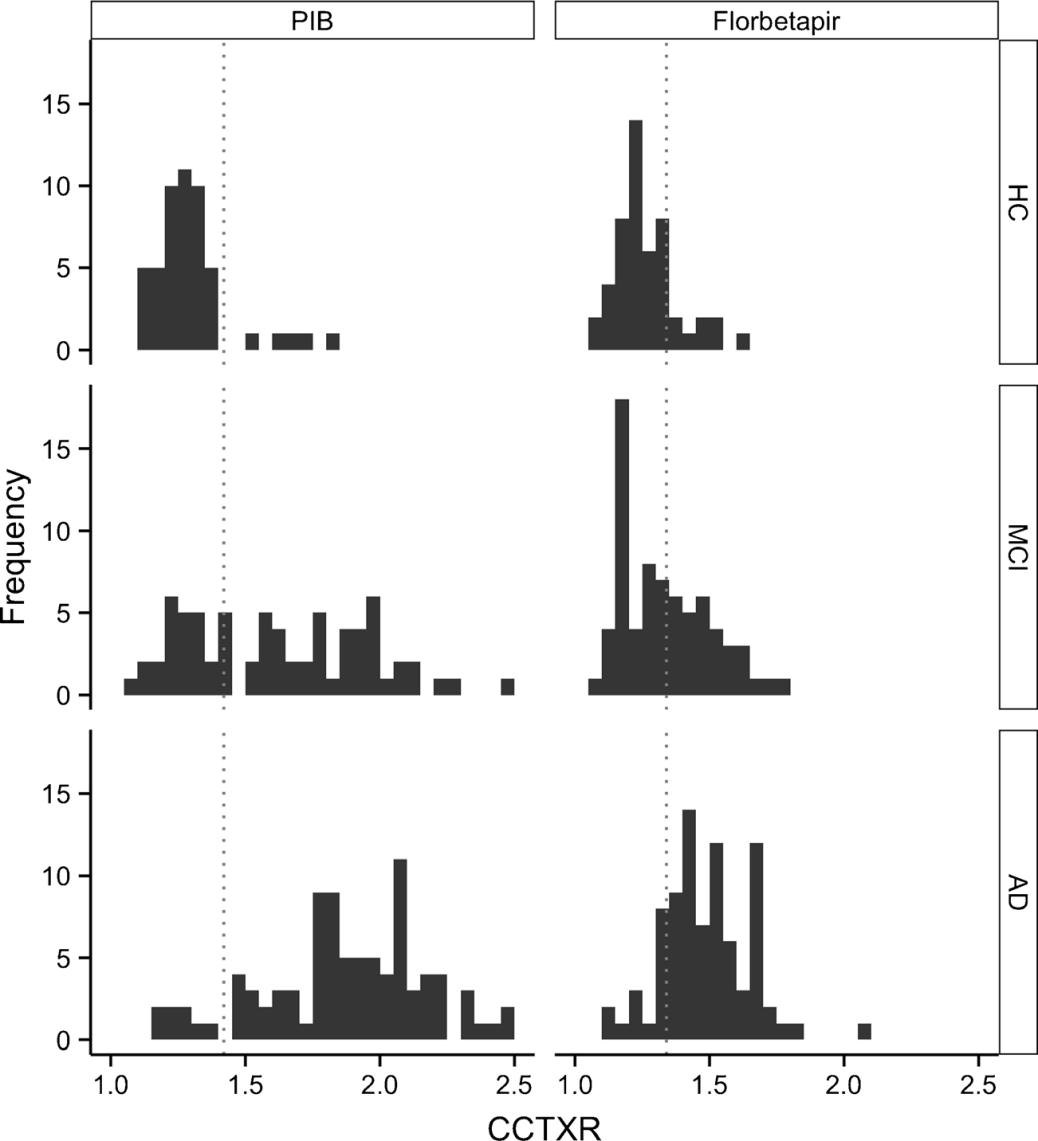
Histogram representing the CCTXR values in HC, MCI and AD patients for [11C]PIB and [18F]Florbetapir. The dotted line in each plot represents the calculated [11C]PIB and [18F]Florbetapir cut-off points (1.42 and 1.34, respectively)

Florbetapir exhibited higher non-specific white matter (WM) relative to GM uptake compared to PIB in the visual inspection of the scans (Fig. 1). The range of CCTXR values were lower and narrower for Florbetapir (0.99–2.07) than for PIB (1.09–2.67) (Fig. 2).

#### Discriminative ability

Florbetapir was less able than PIB to discriminate between HC and AD patients according to the ROC analysis of the CCTXR area under the curve (AUC) values (AUC = 0.864 for Florbetapir [95 % confidence interval (CI): 0.798–0.930] and AUC = 0.931 for PIB [95 % CI: 0.888–0.974]). Consequently, the optimum cut-off point for the Florbetapir data displayed lower sensitivity as well as specificity (1.34, 85 and 82 %, respectively) in comparison to the cut-off point established for PIB (1.42, 91 and 90 %, respectively). The number of amyloid-positive individuals (using these cut-off points) from each disease group is shown in Table 1.

Among the individual ROIs that were included in the analysis, discrimination between HC and AD patients was best for both PIB and Florbetapir in the putamen (Table 2).

**Table 2:** The abilities of [11C]PIB and [18 F]Florbetapir to discriminate between HC and patients with AD for all the included ROIs, as assessed by ROC analysis of the CCTXR AUC values

Discriminative ability		
ROIs	[11C]PIB	[18F]Florbetapir
Putamen	0.966 [0.941–0.990]	0.928 [0.880–0.976]
ACC	0.933 [0.891–0.975]	0.847 [0.780–0.915] ↓_6_
Parietal	0.932 [0.889–0.975]	0.859 [0.794–0.925] ↓_5_
PCC	0.929 [0.887–0.971]	0.882 [0.821–0.943] ↑_2_
Frontal	0.925 [0.881–0.970]	0.835 [0.762–0.907] ↓_7_
Temporal	0.924 [0.877–0.971]	0.875 [0.812–0.939] ↑_3_
Occipital	0.915 [0.867–0.964]	0.871 [0.810–0.931] ↑_4_
Insula	0.908 [0.856–0.959]	0.772 [0.691–0.852]
Caudate Nucleus	0.863 [0.799–0.926]	0.693 [0.606–0.781]
Parahippocampal gyrus	0.834 [0.768–0.899]	0.617 [0.522–0.711]
Thalamus	0.730 [0.648–0.812]	0.415 [0.319–0.511] ↓_12_
Hippocampus	0.553 [0.459–0.647]	0.483 [0.386–0.580] ↑_11_

The results are presented as ROC AUC values [95 % confidence intervals] and the ROIs are ranked in descending order according to the relevant discriminative ability of [11C]PIB. Arrows in the [18F]Florbetapir column represent the difference in ranking from [11C]PIB. (*ACC* anterior cingulate cortex; *PCC* posterior cingulate cortex)

#### Uptake of PIB and Florbetapir is highly correlated

**Fig. 3:**
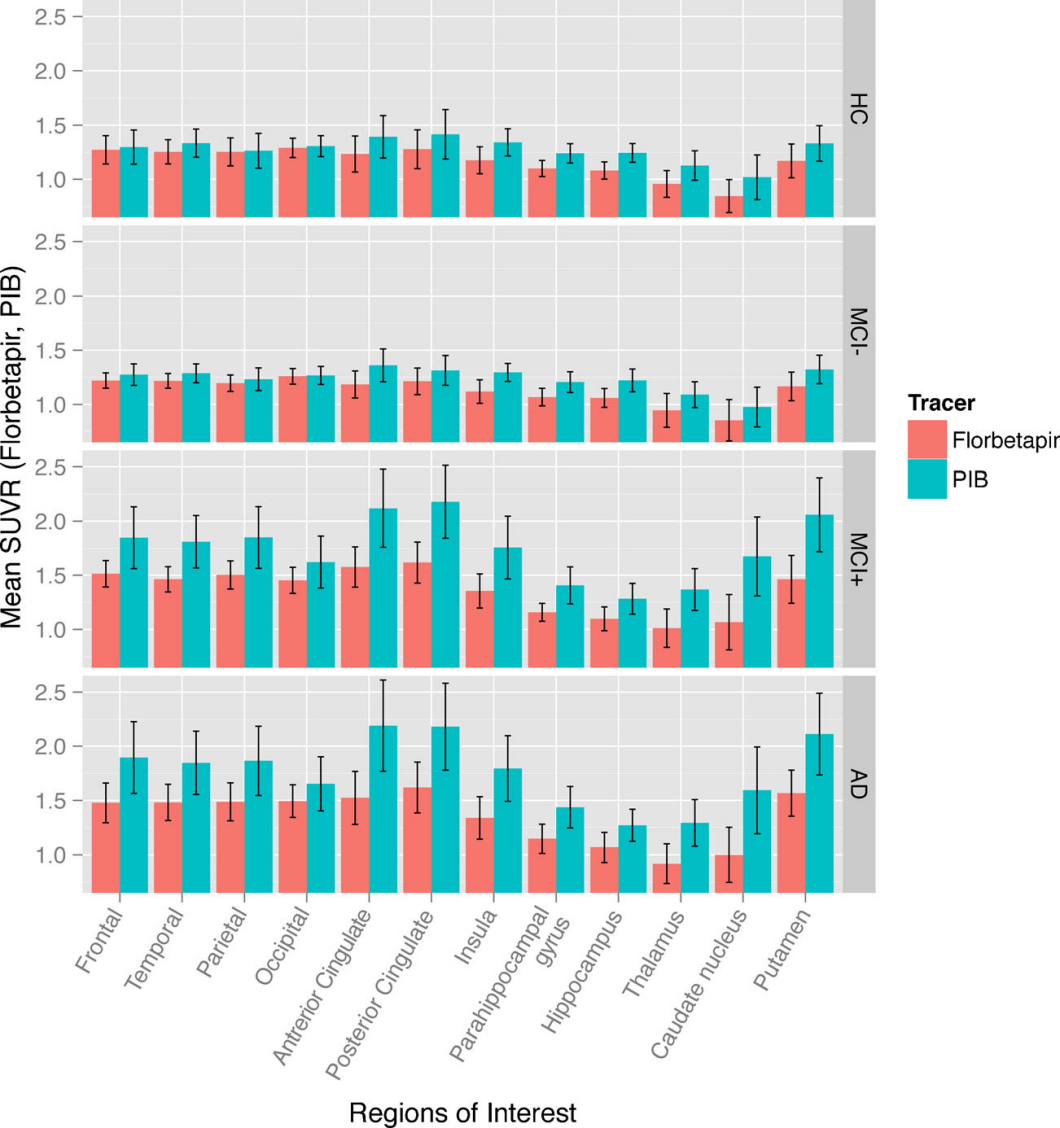
Means ± SDs of tracers’ SUVR values for the investigated ROIs in the respective diagnostic groups; HC, and MCI amyloid-negative (MCI-), MCI amyloid-positive (MCI+) and AD patients. Amyloid positivity has been defined as a CCTXR value above the cut-off points of 1.42 for [11C]PIB and 1.34 for [18F]Florbetapir. The data represented graphically here are tabulated (means ± SDs) in Supplementary Table 2. ([18F]Florbetapir = coral; [11C]PIB = turquoise)

**Fig. 4:**
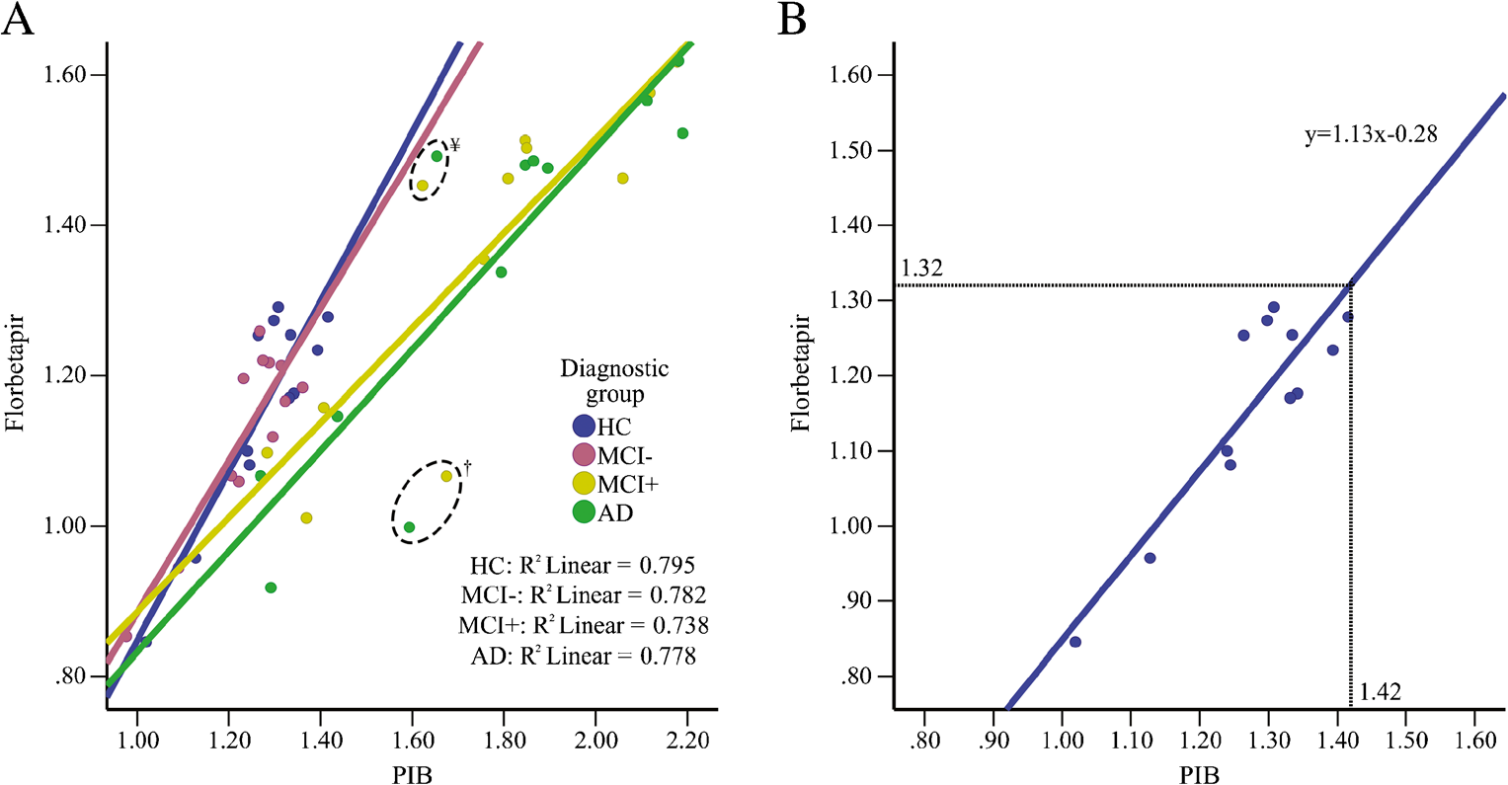
Scatterplots representing the mean uptake ratios (SUVRs) of each ROI for [11C]PIB versus [18F]Florbetapir in the respective groups; (**a**) HC (amyloid-positive and amyloid-negative individuals), and MCI amyloid-negative (MCI-), MCI amyloid-positive (MCI+) and AD patients; and (**b**) the same graph individually for the HC group (amyloid-positive and amyloid-negative individuals). The linear equation derived from the correlation in the HC group (**b**) was used to convert the [11C]PIB cut-off point (1.42) to a [18F]Florbetapir cut-off point (1.32). Amyloid positivity was defined as a CCTXR value above the cut-off points of 1.42 for [11C]PIB and 1.34 for [18F]Florbetapir. Every data point represents the mean value for a bilateral ROI and not an individual participant. †: Caudate nucleus; ¥: Occipital cortex

The MCI group was split into amyloid-negative and amyloid-positive individuals with respect to the above cut-off points for PIB and Florbetapir. The mean SUVR values for the investigated ROIs in the respective diagnostic groups (HC/MCI amyloid-negative/MCI amyloid-positive/AD) followed the same pattern for both tracers (Fig. 3; Supplementary Table 2). The mean regional SUVR values for PIB and Florbetapir across the ROIs examined (*n* = 12) were significantly correlated in the HC (ρ = 0.671, *p* = 0.02, R^2^ = 0.795), MCI amyloid-positive patient (ρ = 0.902, *p* < 0.001, R^2^ = 0.738) and AD patient (ρ = 0.853, *p* < 0.001, R^2^ = 0.778) groups, but not in the MCI amyloid-negative patient groups although a clear trend was observed (ρ = 0.531, *p* = 0.08, R^2^ = 0.782) (Fig. 4a).

Among the disease groups that had a correlation between the two tracers, the HC group had the highest coefficient of determination (R^2^ = 0.795). Therefore, with the use of the linear equation derived from the above correlation (y = 1.13x–0.28), the PIB CCTXR cut-off point (1.42) corresponded to a Florbetapir cut-off point of 1.32 (Fig. 4b).

### Comparison of Florbetapir uptake in different age groups (ADNI data)

#### Demographics

The HC, MCI and AD patients from both age groups were matched for gender and general cognitive status (MMSE). The distribution of the ApoE4 allele differed between the two age groups for the MCI and AD patients but not for the HCs; the younger MCI and AD patients were more likely than the older patients in these groups to be ApoE4 carriers. Moreover, the younger MCI group was significantly more educated than the older group (Table 3).

**Table 3:** Demographics for the within-population (ADNI) age comparison

	Younger ADNI group (55-75 y, *n* = 363)			Older ADNI group (76-93 y, *n* = 363)		
	HC	MCI	AD	HC	MCI	AD
n	123	171	69	123	171	69
Age (years)^a^	70.6 ± 3.1	71.8 ± 2.3	69.0 ± 5.3	81.0 ± 4.0	80.7 ± 3.7	81.4 ± 4.0
Gender (m/f)	55/68	105/66	35/34	66/57	106/65	45/24
Education data available, n (years ± SD)	123 (16.3 ± 2.7)	171 (16.3 ± 2.7^b^)	69 (15.9 ± 2.6)	123 (16.4 ± 2.8)	171 (15.6 ± 2.9^b^)	69 (15.7 ± 2.8)
ApoE status available, n (E4 non-carriers, carriers)	121 (84, 37)	170 (81, 89)^c^	67 (14, 53)^d^	123 (95, 28)	169 (106, 63)^c^	65 (27, 38)^d^
MMSE data available, n (mean score ± SD)	123 (29.1 ± 1.1)	171 (27.9 ± 1.8)	69 (22.6 ± 3.4)	123 (29.0 ± 1.2)	171 (27.7 ± 1.7)	69 (22.3 ± 2.7)
Amyloid Positive, n (%)	24 (20)	83 (49)	56 (81)	37 (30)	91 (53)	50 (72)

Amyloid positivity has been defined as a CCTXR value above the cut-off point of 1.34 for [18F]Florbetapir. (*AD* Alzheimer’s disease; SD standard deviation)

^a^ The mean ages of the two age groups differed significantly for each diagnostic group (*p* < 0.001)

^b^ The younger MCI patients were significantly more educated than the older MCI patients (*p* = 0.029)

^c^ The younger MCI patient group had significantly more ApoE4 carriers than the older MCI group (*p* = 0.005)

^d^ The younger AD patient group had significantly more ApoE4 carriers than the older AD group (*p* = 0.010)

#### Discriminative ability

The application of the Florbetapir cut-off point (1.34) to the two age groups resulted in a higher proportion of amyloid-positive HCs and a lower proportion of amyloid-positive AD patients in the older group than in the younger one. Amyloid positivity occurred to a similar extent in MCI patients in the two age groups (Table 3). Therefore, the sensitivity and specificity of Florbetapir in discriminating between HC and AD patients were poorer in the older ADNI group (72 and 70 %, respectively) than in the younger group (81 and 80 %, respectively).

#### Amyloid-negative AD patients

The amyloid-negative AD patients (*n* = 32) were less likely to be ApoE4 carriers relative to the amyloid-positive patients (*n* = 12 [38 %] vs *n* = 79 [79 %], *p* < 0.001). No difference was observed between the two groups with respect to MMSE or years of education.

In the visual assessment of the FDG-PET scans in the amyloid-negative AD patient group the hypometabolism was regionally diffuse. The agreement between the raters was poor and not significant (41 %) with both raters consistently assessing nine individuals (28 %) of the amyloid-negative AD patient group as positive for an AD-like pattern of hypometabolism in their FDG scans. No difference was observed between the young and the old subgroups.

### Florbetapir uptake in the different diagnostic groups

#### Healthy controls

Two-way ANOVA indicated that older age, independent of ApoE4 status, had a significant positive effect on amyloid deposition only in the putamen (F_1,240_ = 14.34, *p* < 0.001) and a negative effect in the thalamus (F_1,240_ = 5.10, *p* = 0.025) in HCs. The presence of the ApoE4 allele had an independent, significant, positive effect on amyloid deposition in every ROI examined except the hippocampus and parahippocampal gyrus (Table 4). No statistically significant interaction was observed between age group and ApoE4 carrier status in any of the ROIs examined.

**Table 4:** The independent effects of age and ApoE4 carrier status on the [18 F]Florbetapir uptake ratio for the examined ROI (two-way ANOVA analysis)

Disease group	Healthy Controls (HCs)				Alzheimer’s Disease (AD) patients			
	Younger HCs (55-75 y)	Older HCs (76-93 y)	p^a^	p^b^	Younger AD (55-75 y)	Older AD (76-93 y)	p^a^	p^b^
CCTXR	1.26 ± 0.12	1.26 ± 0.17	0.646	<0.001	1.48 ± 0.17	1.39 ± 0.21	0.036	<0.001
Frontal	1.27 ± 0.13	1.25 ± 0.18	0.755	0.001	1.48 ± 0.18	1.38 ± 0.22	0.040	<0.001
Temporal	1.26 ± 0.11	1.27 ± 0.15	0.196	<0.001	1.48 ± 0.16	1.41 ± 0.20	0.069	<0.001
Parietal	1.26 ± 0.13	1.25 ± 0.17	0.967	0.001	1.49 ± 0.18	1.40 ± 0.21	0.029	<0.001
Occipital	1.31 ± 0.10	1.32 ± 0.13	0.355	0.020	1.49 ± 0.16	1.46 ± 0.17	0.255	0.005
ACC	1.22 ± 0.18	1.21 ± 0.23	0.626	<0.001	1.53 ± 0.25	1.39 ± 0.27	0.028	<0.001
PCC	1.26 ± 0.18	1.29 ± 0.23	0.155	<0.001	1.62 ± 0.25	1.51 ± 0.28	0.041	<0.001
Insula	1.15 ± 0.12	1.13 ± 0.17	0.992	0.002	1.35 ± 0.20	1.22 ± 0.21	0.005	<0.001
Caudate nucleus	0.83 ± 0.17	0.79 ± 0.21	0.338	<0.001	1.00 ± 0.25	0.87 ± 0.30	0.075	0.017
Putamen	1.17 ± 0.14	1.23 ± 0.17	<0.001	<0.001	1.57 ± 0.23	1.53 ± 0.24	0.832	<0.001
Thalamus	0.94 ± 0.12	0.88 ± 0.15	0.025	<0.001	0.92 ± 0.19	0.82 ± 0.16	0.032	0.157
Parahippocampal gyrus	1.09 ± 0.08	1.09 ± 0.10	0.433	0.055	1.15 ± 0.13	1.10 ± 0.13	0.054	0.031
Hippocampus	1.08 ± 0.08	1.06 ± 0.10	0.259	0.725	1.07 ± 0.12	0.99 ± 0.16	0.004	0.311
ApoE4 carriers, n (%)	37 (31)	28 (23)			53 (79)^c^	38 (58)^c^		

*ACC* anterior cingulate cortex; *PCC* posterior cingulate cortex

^a^ Independent effects of age on the amyloid load in the ROIs after inclusion of ApoE4 status

^b^ Independent effects of ApoE4 status on the amyloid load in the ROIs after inclusion of age group

^c^ Significantly more younger than older Alzheimer’s disease patients were ApoE4 carriers (*p* = 0.010)

**Fig. 5:**
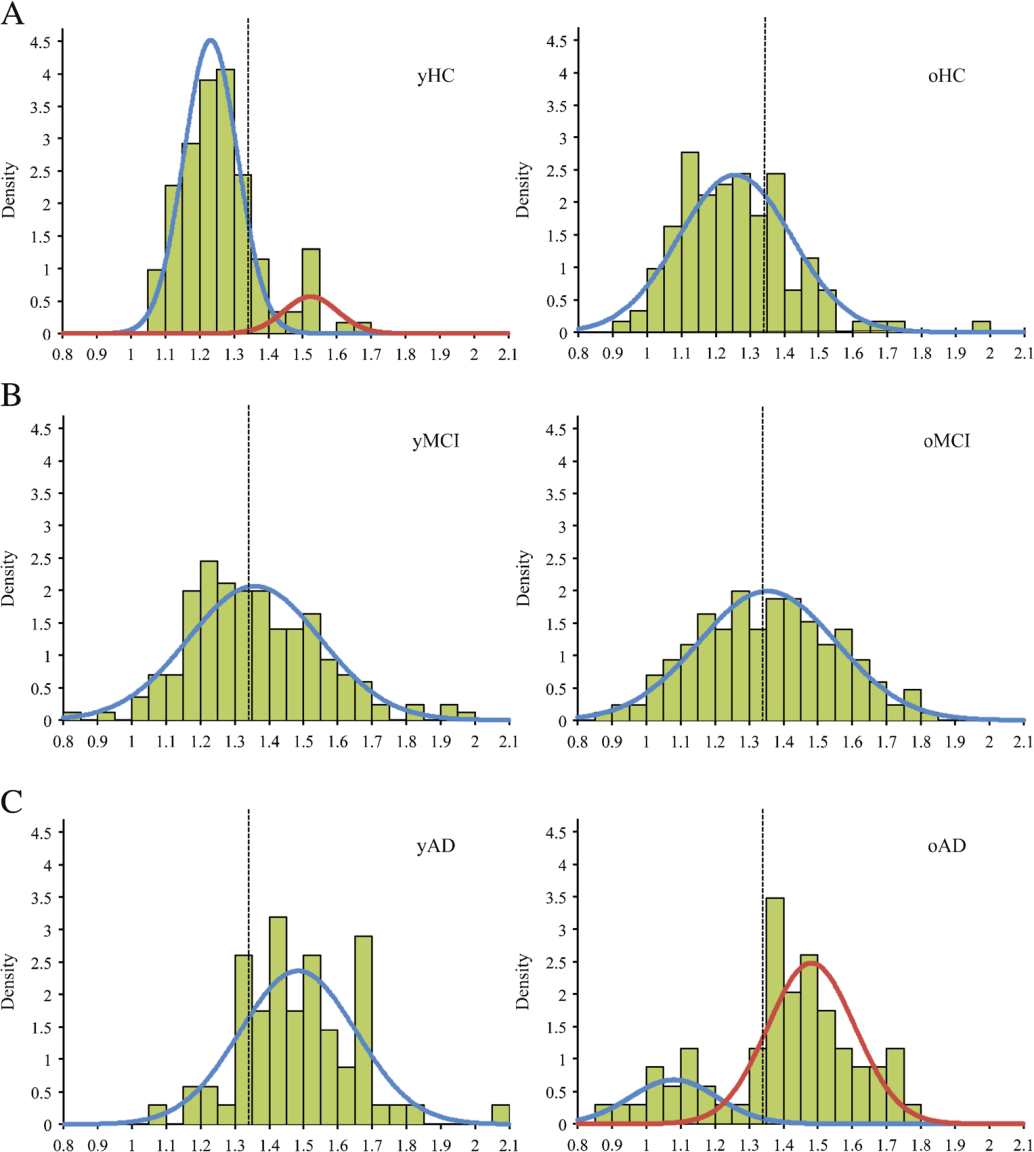
Density plots of the [18F]Florbetapir CCTXR values of younger (y; 55–75 y) and older (o; 76–93 y) age groups in (**a**) HCs, (**b**) MCI patients and (**c**) AD patients. Mixture model analysis using the expectation-maximisation algorithm allowed investigation of the possible underlying components of the distributions. The *dotted line* in each plot represents the calculated [18F]Florbetapir cut-off point (1.34)

The Kolmogorov-Smirnov test revealed that the distribution of the CCTXR results in younger HCs differed significantly from a normal distribution pattern (*p* = 0.002), whereas the distribution in older HCs was more likely to follow a normal distribution pattern (*p* = 0.083). Mixture model analysis found that the CCTXR values for the younger HCs were more likely to be a mixture of two components with mean values of 1.23 ± 0.08 (*n* = 110) and 1.52 ± 0.08 (*n* = 13), representing relatively low and high amyloid deposition, respectively. The values for the older HCs were more likely to follow a single normal distribution, with a mean value of 1.26 ± 0.16 (*n* = 123) (Fig. 5a). There were no differences between the younger HCs in the higher and lower amyloid deposition groups with respect to MMSE, years of education, age or gender distribution. However, the younger HCs in the higher amyloid deposition group were more likely to be ApoE4 carriers than those in the lower amyloid deposition group (*n* = 9 [69 %] vs *n* = 28 [26 %], *p* = 0.001).

#### AD patients

In AD patients, two-way ANOVA analysis indicated a significant negative effect of older age, independent of the presence of the ApoE4 allele, on the levels of amyloid deposition in diffuse cortical and subcortical ROIs: CCTXR (F_1,128_ = 4.47, *p* = 0.036), frontal (F_1,128_ = 4.29, *p* = 0.040), parietal cortex (F_1,128_ = 4.89, *p* = 0.029), anterior cingulate (F_1,128_ = 4.96, *p* = 0.028), posterior cingulate (F_1,128_ = 4.28, *p* = 0.041), insular cortex (F_1,128_ = 8.20, *p* = 0.005), thalamus (F_1,128_ = 4.71, *p* = 0.032) and hippocampus (F_1,128_ = 8.46, *p* = 0.004). The independent, positive effect of the ApoE4 allele on amyloid distribution was significant in every ROI examined except the thalamus and the hippocampus (Table 4). No statistically significant interaction was observed between age group and ApoE4 carrier status in any of the ROIs examined except the thalamus (F_1,128_ = 4.74, *p* = 0.035); the presence of the ApoE4 allele increased the amyloid levels in younger AD patients but not in the older group.

The Kolmogorov-Smirnov test revealed that the distribution of the CCTXR results in younger AD patients was more likely to follow a normal distribution pattern (*p* = 0.200) whereas the values for the older AD patients differed significantly from a normal distribution pattern (*p* = 0.007). Mixture model analysis showed that the CCTXR values for the younger AD patients were more likely to follow a single normal distribution, with a mean value of 1.48 ± 0.17 (*n* = 69). The values for the older AD patients were more likely to follow a mixed distribution containing two components with mean values of 1.08 ± 0.13 (*n* = 15) and 1.48 ± 0.13 (*n* = 54), representing relatively low and high amyloid deposition levels, respectively (Fig. 5c). There were no differences between the higher and lower amyloid deposition groups in the older AD patients with respect to MMSE, age or gender distribution. However, the older AD patients in the lower amyloid deposition group were less likely to be ApoE4 carriers (*n* = 1 [7 %] vs *n* = 37 [74 %], *p* < 0.001) and more likely to be highly educated (17.1 ± 2.5 vs 15.4 ± 2.8 y, *p* = 0.036) than those in the higher amyloid deposition group.

Two-way ANOVA analysis of the younger AD patients in comparison to the older AD patients with high amyloid deposition, indicated a significant positive effect of older age, independent of the presence of the ApoE4 allele, on the levels of amyloid deposition only in the putamen (F_1,101_ = 4.61, *p* = 0.034).

#### MCI patients

Two-way ANOVA analysis indicated a significant positive effect of older age, independent of the presence of the ApoE4 allele, on the levels of amyloid deposition in the posterior cingulate (F_1,335_ = 4.31, *p* = 0.039), and the putamen (F_1,335_ = 5.78, *p* = 0.017). The independent positive effect of the ApoE4 allele on amyloid distribution was significant in every ROI examined (Supplementary Table 3).

The Kolmogorov-Smirnov test revealed that the amyloid distributions for both younger and older MCI patients did not differ significantly from a normal distribution pattern (*p* = 0.068 and *p* = 0.200, respectively). Mixture model analysis confirmed that the distributions of both younger and older MCI patients were more likely to follow a single normal distribution, with mean values of 1.36 ± 0.19 (*n* = 171) for younger MCI patients and 1.35 ± 0.20 (*n* = 171) for older MCI patients (Fig. 5b).

## Discussion

Although the recently developed [18F] amyloid-PET tracers bind to the same high affinity site as PIB [36], further investigation was required into the comparability of the tracers and the extent of inter- and intra-cohort variation in the relevant cohorts.

The first aim of this study was to compare two age- and sex-matched populations, which were unrelated, geographically distinct and scanned with different amyloid tracers (PIB in the DiMI cohort and Florbetapir in the ADNI cohort).

This study illustrates the high agreement of regional uptake as well as correlation between the two tracers across the different ROIs examined in every diagnostic group, although different individuals were scanned with each tracer. The lack of a clear correlation, however, in the MCI amyloid-negative group probably reflects the extensive heterogeneity of the pathology in that specific group.

Despite the significant correlation, Florbetapir performed less well at discriminating between HC and AD patients than PIB. Florbetapir binds less than PIB to fibrillar Aβ in GM and has relatively more non-specific WM uptake on visual inspection, as previously reported by Wolk et al. [12]. Data from Landau et al. [11] also supports this, in that PIB displayed higher GM uptake than Florbetapir, although the two tracers exhibited comparable WM uptake. Florbetapir is also known, from animal studies, to have lipophilic metabolites that can cross the blood–brain barrier and increase the non-specific background signal in amyloid PET [37], in comparison to PIB [3]. These factors fit with the current Florbetapir data in our study, since the GM range for Florbetapir uptake was much narrower than that for PIB. Moreover, the difference in the range of the two tracers becomes especially apparent in high SUVR values, where PIB displays a much wider range in comparison to Florbetapir, whereas in low SUVR values the ranges of the two tracers are similar (Fig. 2). This difference in range has probably led to a steeper slope of the correlation between tracers in the AD and MCI amyloid-positive groups in comparison to the HC and MCI amyloid-negative groups.

As discussed above, the relatively higher non-specific WM uptake (and metabolites) of Florbetapir and, therefore, the greater spill over from WM into GM in comparison to PIB could have affected the effect size between HC and AD patients, despite the observed correlation. In the current study, no partial volume correction was applied to either the PIB or Florbetapir data in order to further illustrate the robust comparability between the two tracers regardless of their different characteristics. Nonetheless, in order to account for the spill over of non-specific WM binding to GM with Florbetapir we investigated the use of different reference regions, as the cerebellar GM reference used for the PIB data was sub-optimum for the Florbetapir data. A reference region containing partly WM (whole cerebellum) at least partly cancelled out the spill over from the highly variable non-specific WM binding of Florbetapir, resulting in reduced variability in the HC group and an increase in the effect size between HC and AD patients.

Neocortical cut-off points for amyloid positivity were defined in order to examine the sensitivity and specificity of each tracer. The CCTXR cut-off point (1.42) and sensitivity/specificity established here for PIB were comparable to values in the literature [16, 26, 38, 39]. Conversely, the cut-off point for Florbetapir (1.34) was higher than the range of previously published values (1.08–1.30) [17, 40–42], although it was very close to the cut-off of the study that used a methodology similar to the current one (1.30) [41]. However, despite the difference in the absolute cut-off value, Florbetapir detected similar percentages of amyloid-positive HC and AD patients in a similar relatively young age group [17]. Moreover, use of the linear correlation between the two tracers in the HC group allowed us to show that the CCTXR cut-off point for PIB (1.42) could be converted to a Florbetapir cut-off point (1.32) with high agreement to the value derived from the independent ROC analysis of the Florbetapir data (1.34). Interestingly, we observed that although the cut-off value did not change significantly, there was a difference in the discriminative abilities of the two Florbetapir cut-off points (89/76 and 85/82, respectively), probably due to the smaller range of the Florbetapir values.

The large variance in the published cut-off points poses questions regarding the use of amyloid PET cut-offs in a clinical setting. Cut-off points are known to be sensitive to the specific tracer for which they were generated and the methodology implemented, further limiting the wide use of a single value. However because of the inherent problems with cut-off values, there have been recent efforts to standardise quantitative amyloid imaging measures on a centiloid scale (0–100) for all amyloid tracers ([11C] and [18 F]). Such standardisation will enable different researchers using different amyloid PET tracers to compare their data more easily [43], particularly when measuring treatment effects in clinical trials. Nonetheless, the combination of different amyloid PET tracers on the same scale has important caveats that are illustrated in this study. Although the tracers studied are comparable, their different inherent characteristics (non-specific WM binding, range of GM values, discriminative ability of the two tracers) limit their interchangeable use. Moreover, according to the original centiloid paper [43], calibrating the values of different tracers onto a common scale is not lacking other limitations. As it is illustrated in that study, at least 25 individuals should have been scanned with both PIB as well as the other amyloid-specific tracers in an interval of less than 3 months in order for each centre to convert the values of the latter tracers into centiloids. Very few centres worldwide have such complimentary amyloid PET data. Although one of the goals of the centiloid project is to encourage centres that do have the data to make the scaling conversions possible from “non-standard” tracers available for all, the data for the non-standard scaling conversions are, as yet, unavailable to the wider PET community.

The best discrimination between HC and AD patients among the investigated ROIs in our study was observed in the putamen. It is already known from presenilin-1 mutation carriers that Aβ deposition in the striatum is an early phenomenon in the progression of autosomal-dominant AD [44]. However, it is believed that Aβ accumulates in the striatum relatively late in the course of sporadic AD pathology [2, 45]. Our findings are supported by autopsy studies in which the presence of moderately frequent/frequent striatal plaques in the putamen was used to differentiate between the presence and absence of clinicopathological AD with a sensitivity and specificity above 85 % [46] and by a machine-learning amyloid PET study where the voxels with the highest feature weights for classification between HC and AD patients were located in the striatum [47]. Therefore, clinical assessment of subcortical structures for the presence of Aβ pathology could be of great importance in early AD diagnosis.

The difference between the two geographically distinct recruitment cohorts (North American and European) in the total years of education exceeded 3 years in every disease group. The average educational attainment of the individuals in the North American cohort (ADNI) was high, equivalent to university graduate, whereas the average participant in the European cohort (DiMI) was equivalent to high-school graduate. It has already been shown that education affects the relationship between cognition and the pathological burden of AD [48]. While the mechanism for this phenomenon is not completely understood the differences in the populations from which participants are recruited should be taken into account as they could create discrepancies in the observed results.

The second objective of this paper was to investigate the effect of age on amyloid PET data from within the large ADNI population sample. The younger HCs had significantly less amyloid deposition than the older HCs only in the putamen, independently of their ApoE4 status, whereas no significant differences in deposition were observed in the composite neocortical region in younger or older HCs. The composite cortical uptake values, however, for the younger HCs appeared to be distinctly different for amyloid-positive and amyloid-negative subjects. Conversely, probably due to the age-related accumulation of amyloid [49], distribution in the older HCs was more likely to be unimodal, shifted to the amyloid-positive side, without any clear border between amyloid-positive and amyloid-negative individuals.

Although most of the research investigating the role of age in the amyloid load of AD patients has focused on EoAD (onset at age <65 y) and LoAD (onset at age >65 y) [23–25], we decided to investigate two groups of older patients in order to explore the effect of age on the amyloid load in a more elderly population. In our study, age had an independent effect on the amyloid load in diffuse cortical and subcortical ROIs; amyloid deposition was lower in the older AD patients than in the younger AD group (i.e., those aged <75 y). Differences between the age groups were also found in the distribution of amyloid in the composite cortical regions in AD patients. The distribution of amyloid in older patients was more likely to be a mixture of two normal distributions, with a clear distinction between amyloid-positive and amyloid-negative patients, whereas the distribution in younger AD patients was unimodal. When we investigated the true AD groups (high amyloid uptake), age related differences between groups were observed only in the putamen, where young AD patients showed significantly lower amyloid deposition in comparison to the older AD patients. This effect was observed in all diagnostic groups (HC, MCI, AD), supporting an important role of the striatum in the age-related accumulation of Aβ.

Although a large number of studies have investigated the discriminative ability of amyloid imaging in individual cohorts, no other study to our knowledge has been designed to define and compare the additive value of amyloid imaging in the assessment of patients across different age groups. The results of this study illustrate the diminished sensitivity and specificity of the Florbetapir cut-off point in an advanced elderly population in comparison to that in younger individuals, when the clinical diagnosis was used as the gold standard, and particularly the NINCDS-ARDRA criteria [27]. The high number of amyloid-negative AD patients of advanced age in the ADNI population, however, poses questions regarding the diagnostic assessment of these patients, especially since recent studies have shown that these individuals do not tend to deteriorate neuropsychologically [50], which probably indicates a population of patients who have been misdiagnosed as having typical AD. The amyloid-negative patients investigated here were more likely to be ApoE4 non-carriers and on the whole had inconclusive FDG-PET scans, due to diffuse non-specific reductions in FDG uptake. Overall, in these patients, two biomarkers and one genetic marker suggest that the cause of their dementia is unlikely to be related to AD pathology and, consequently, they might have been misdiagnosed. Conclusively, the incorporation of amyloid PET into the clinical assessment process could have led to reclassification of the amyloid-negative demented patients, for instance, as suspected non-amyloid-pathology dementia, which would have obvious therapeutic implications. The effect of reclassification of clinically diagnosed AD patients with the use of amyloid PET imaging was found especially evident in the elderly patient population (30 % amyloid negative), where an additional 10 % of demented patients would benefit in comparison to the group aged younger by an average of 10 years (20 % amyloid negative).

The independent positive effect of the ApoE4 allele on the amyloid load was significant in both HC and AD patients in every ROI except the hippocampus. This effect of ApoE4 on the amyloid load in healthy individuals is in line with previous publications [16, 51, 52]. Although the results in AD patients differ from those in some published studies [53–55], they are consistent with others [56, 57]. The lack of an ApoE4 effect in the hippocampus could be associated with the paucity of neuritic plaques in this area [1, 58–60], or the form of Aβ that can be detected with the available PET amyloid tracers.

In summary, these results illustrate good correlation between two amyloid-specific radiotracers, Florbetapir and PIB, in detecting Aβ deposition in unrelated, matched patient populations. The results suggest that these tracers are comparable, not only in the same participants but also across different cohorts, where different population characteristics and protocols apply. Nonetheless, the inherent characteristics of the tracers vary substantially (non-specific WM binding, range of GM values, discriminative ability of the two tracers), limiting the interchangeable use of the tracers in multicentre studies. Notwithstanding its limitations, this study also suggests that the role of amyloid PET imaging becomes increasingly important with increasing age in the diagnostic assessment of clinically impaired patients. This finding could lead to a dramatic decrease of the routine false AD diagnoses in very old patients suffering from memory complaints with important therapeutic implications.

## Electronic supplementary material

Supplemental Table 1 (DOCX 15 kb)

Supplemental Table 2 (DOCX 18 kb)

Supplemental Table 3 (DOCX 16 kb)
