# Supplementary material for: Amyloid PET in European and North American cohorts; and exploring age as a limit to clinical use of amyloid imaging
Source: Eur J Nucl Med Mol Imaging. 2015 Jul 2;42(10):1492–506. doi: 10.1007/s00259-015-3115-5 (PMC4521094; doi:10.1007/s00259-015-3115-5)
Supplement: Supplementary file 1 — (DOCX 15 kb) [file 259_2015_3115_MOESM1_ESM.docx]

Supplementary Table 1. Comparison of the reference regions used for intensity normalisation and creation of the respective SUVR [18F]Florbetapir images for each individual.

|  | Reference regions for Florbetapir data | | |
| --- | --- | --- | --- |
|  | Median value for the cerebellar GM | Median value for the whole cerebellum | Mean value for the whole cerebellum |
| Variance of HC (n=51) | 0.025 | 0.022 | **0.014** |
| Effect size between HC (n=51) and AD patients (n=84) | -1.027 | -1.094 | **-1.481** |

When the mean value for the whole cerebellum was used as a reference region the CCTXR exhibited the lowest variance in the HC group and the highest effect size between HC and Alzheimer’s disease (AD) patients, examined with the use of Cohen’s d.
