# Supplementary material for: Amyloid PET in European and North American cohorts; and exploring age as a limit to clinical use of amyloid imaging
Source: Eur J Nucl Med Mol Imaging. 2015 Jul 2;42(10):1492–506. doi: 10.1007/s00259-015-3115-5 (PMC4521094; doi:10.1007/s00259-015-3115-5)
Supplement: Supplementary file 2 — (DOCX 18 kb) [file 259_2015_3115_MOESM2_ESM.docx]

Supplementary Table 2. Tracer SUVR values for all investigated ROIs in the respective diagnostic groups; HC, and MCI amyloid-negative (MCI-), MCI amyloid-positive (MCI+) and Alzheimer’s disease (AD) patients.

|  | HC | | MCI- | | MCI+ | | AD | |
| --- | --- | --- | --- | --- | --- | --- | --- | --- |
|  | PIB | Florbetapir | PIB | Florbetapir | PIB | Florbetapir | PIB | Florbetapir |
| n | 51 | 51 | 26 | 41 | 46 | 31 | 90 | 84 |
| Frontal | 1.30±0.16 | 1.27±0.13 | 1.27±0.10 | 1.22±0.07 | 1.86±0.28 | 1.51±0.12 | 1.90±0.33 | 1.48±0.18 |
| Temporal | 1.33±0.13 | 1.26±0.11 | 1.30±0.10 | 1.22±0.07 | 1.81±0.24 | 1.47±0.11 | 1.85±0.29 | 1.48±0.17 |
| Parietal | 1.26±0.16 | 1.26±0.13 | 1.24±0.11 | 1.20±0.08 | 1.86±0.28 | 1.51±0.13 | 1.86±0.32 | 1.49±0.17 |
| Occipital | 1.31±0.10 | 1.29±0.09 | 1.28±0.10 | 1.26±0.07 | 1.62±0.24 | 1.45±0.12 | 1.65±0.25 | 1.49±0.15 |
| ACC | 1.39±0.20 | 1.23±0.17 | 1.36±0.15 | 1.19±0.13 | 2.13±0.35 | 1.58±0.19 | 2.19±0.42 | 1.53±0.25 |
| PCC | 1.42±0.23 | 1.28±0.18 | 1.32±0.14 | 1.22±0.12 | 2.19±0.32 | 1.63±0.19 | 2.18±0.40 | 1.63±0.23 |
| Insula | 1.34±0.13 | 1.18±0.12 | 1.30±0.08 | 1.13±0.11 | 1.76±0.29 | 1.36±0.16 | 1.79±0.30 | 1.34±0.19 |
| Caudate nucleus | 1.02±0.21 | 0.84±0.15 | 0.98±0.19 | 0.85±0.19 | 1.68±0.36 | 1.06±0.26 | 1.59±0.40 | 0.99±0.25 |
| Putamen | 1.33±0.16 | 1.17±0.16 | 1.33±0.13 | 1.17±0.13 | 2.07±0.33 | 1.46±0.22 | 2.11±0.38 | 1.57±0.21 |
| Thalamus | 1.13±0.14 | 0.96±0.12 | 1.09±0.12 | 0.94±0.16 | 1.37±0.19 | 1.01±0.18 | 1.29±00.22 | 0.92±0.18 |
| Parahippocampal gyrus | 1.24±0.09 | 1.10±0.07 | 1.21±0.10 | 1.07±0.08 | 1.41±0.17 | 1.16±0.08 | 1.44±0.19 | 1.15±0.13 |
| Hippocampus | 1.24±0.09 | 1.08±0.08 | 1.22±0.10 | 1.06±0.09 | 1.29±0.14 | 1.10±0.11 | 1.27±0.15 | 1.07±0.14 |

Amyloid positivity has been defined as a CCTXR value above the cut-off points of 1.42 for [11C]PIB and 1.34 for [18F]Florbetapir. The SUVR values are presented as means ± standard deviations. (ACC = anterior cingulate cortex; PCC = posterior cingulate cortex)
