# Supplementary material for: Amyloid PET in European and North American cohorts; and exploring age as a limit to clinical use of amyloid imaging
Source: Eur J Nucl Med Mol Imaging. 2015 Jul 2;42(10):1492–506. doi: 10.1007/s00259-015-3115-5 (PMC4521094; doi:10.1007/s00259-015-3115-5)
Supplement: Supplementary file 3 — (DOCX 16 kb) [file 259_2015_3115_MOESM3_ESM.docx]

| **Disease group** | **Mild Cognitive Impairment (MCI) patients** | | | |
| --- | --- | --- | --- | --- |
|  | Younger MCI  (55-75y) | Older MCI  (76-93y) | **p^a^** | **p^b^** |
| **CCTXR** | 1.36±0.19 | 1.35±0.20 | 0.359 | **<0.001** |
| **Frontal** | 1.37±0.21 | 1.35±0.22 | 0.541 | **<0.001** |
| **Temporal** | 1.35±0.18 | 1.35±0.19 | 0.163 | **<0.001** |
| **Parietal** | 1.35±0.20 | 1.35±0.21 | 0.370 | **<0.001** |
| **Occipital** | 1.37±0.15 | 1.39±0.16 | 0.199 | **<0.001** |
| **ACC** | 1.35±0.27 | 1.34±0.29 | 0.425 | **<0.001** |
| **PCC** | 1.40±0.28 | 1.44±0.29 | **0.039** | **<0.001** |
| **Insula** | 1.23±0.20 | 1.19±0.21 | 0.615 | **<0.001** |
| **Caudate nucleus** | 0.90±0.23 | 0.86±0.25 | 0.391 | **<0.001** |
| **Putamen** | 1.35±0.26 | 1.38±0.25 | **0.017** | **<0.001** |
| **Thalamus** | 0.92±0.16 | 0.88±0.17 | 0.208 | **<0.001** |
| **Parahippocampal gyrus** | 1.12±0.13 | 1.11±0.13 | 0.509 | **<0.001** |
| **Hippocampus** | 1.07±0.12 | 1.05±0.12 | 0.294 | **0.005** |
| **ApoE4 carriers, n (%)** | 89 (52) | 63 (37) |  |  |

Supplementary Table 3. The independent effects of age and ApoE4 carrier status on the [18F]Florbetapir SUVR for the examined ROI in the Mild Cognitive Impairment patients (two-way ANOVA analysis).

ACC = anterior cingulate cortex; PCC = posterior cingulate cortex.

^a^ Independent effects of age on the amyloid load in the ROIs after inclusion of ApoE4 status.

^b^ Independent effects of ApoE4 status on the amyloid load in the ROIs after inclusion of age group.

^c^ Significantly more younger than older Mild Cognitive Impairment patients were ApoE4 carriers (p=0.006).
